# Supplementary figures and images for: A Tetratricopeptide Repeat Scaffold Couples Signal Detection to OdhI Phosphorylation in Metabolic Control by the Protein Kinase PknG
Source: mBio. 2021 Oct 5;12(5):e01717-21. doi: 10.1128/mBio.01717-21 (PMC8546545; doi:10.1128/mBio.01717-21)

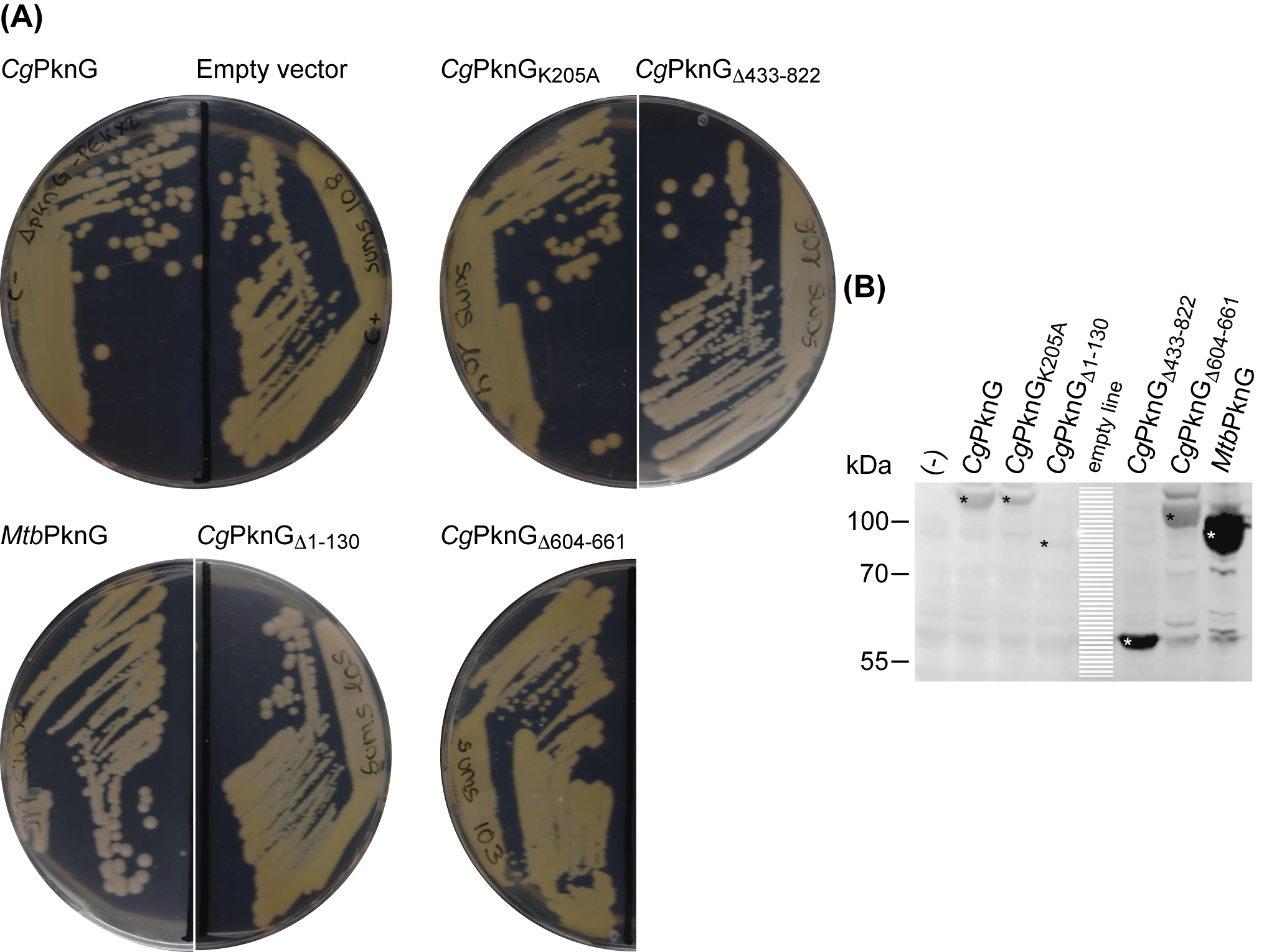

Supplement: FIG S1 [file mbio.01717-21-sf001.tif]

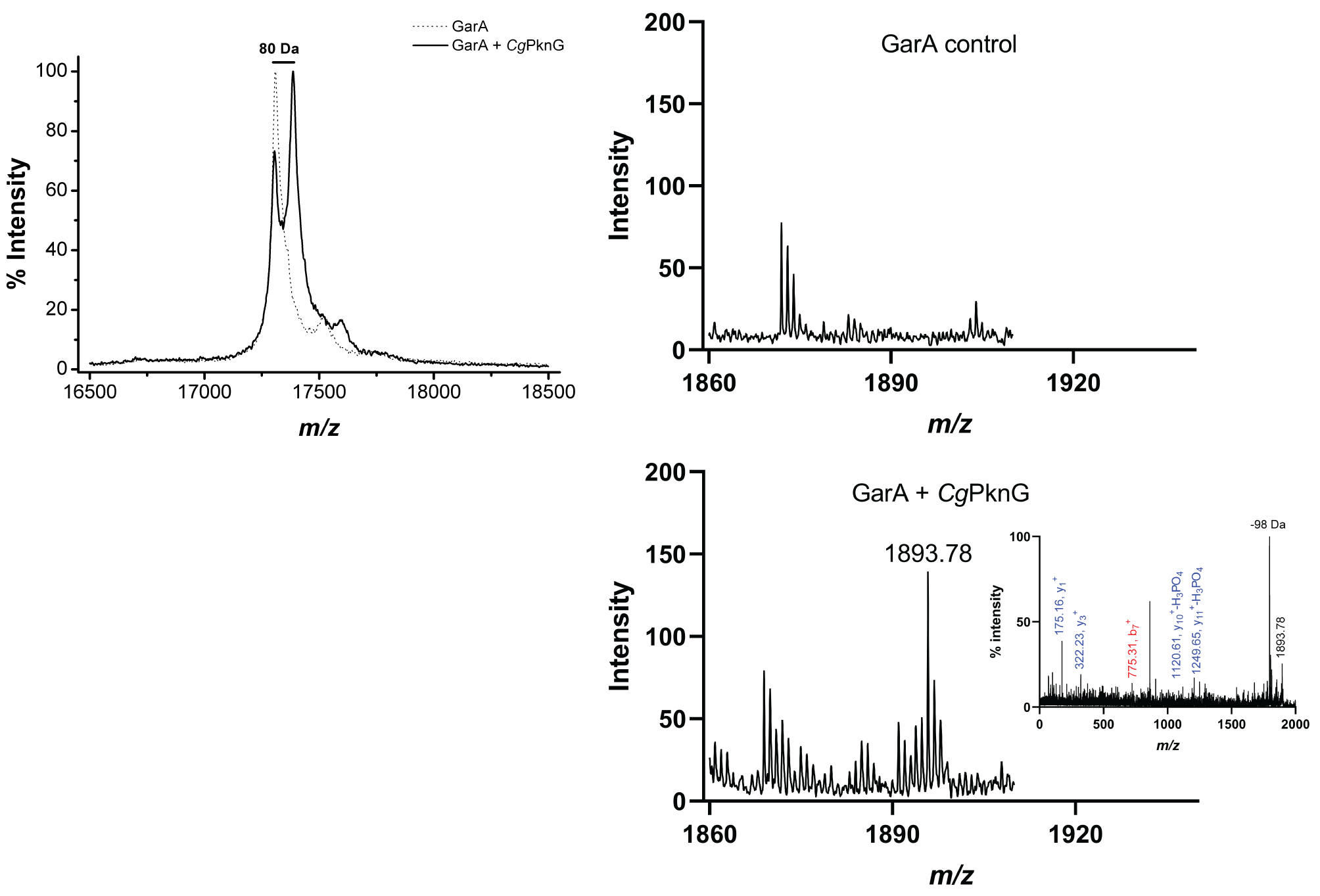

Supplement: FIG S2 [file mbio.01717-21-sf002.tif]

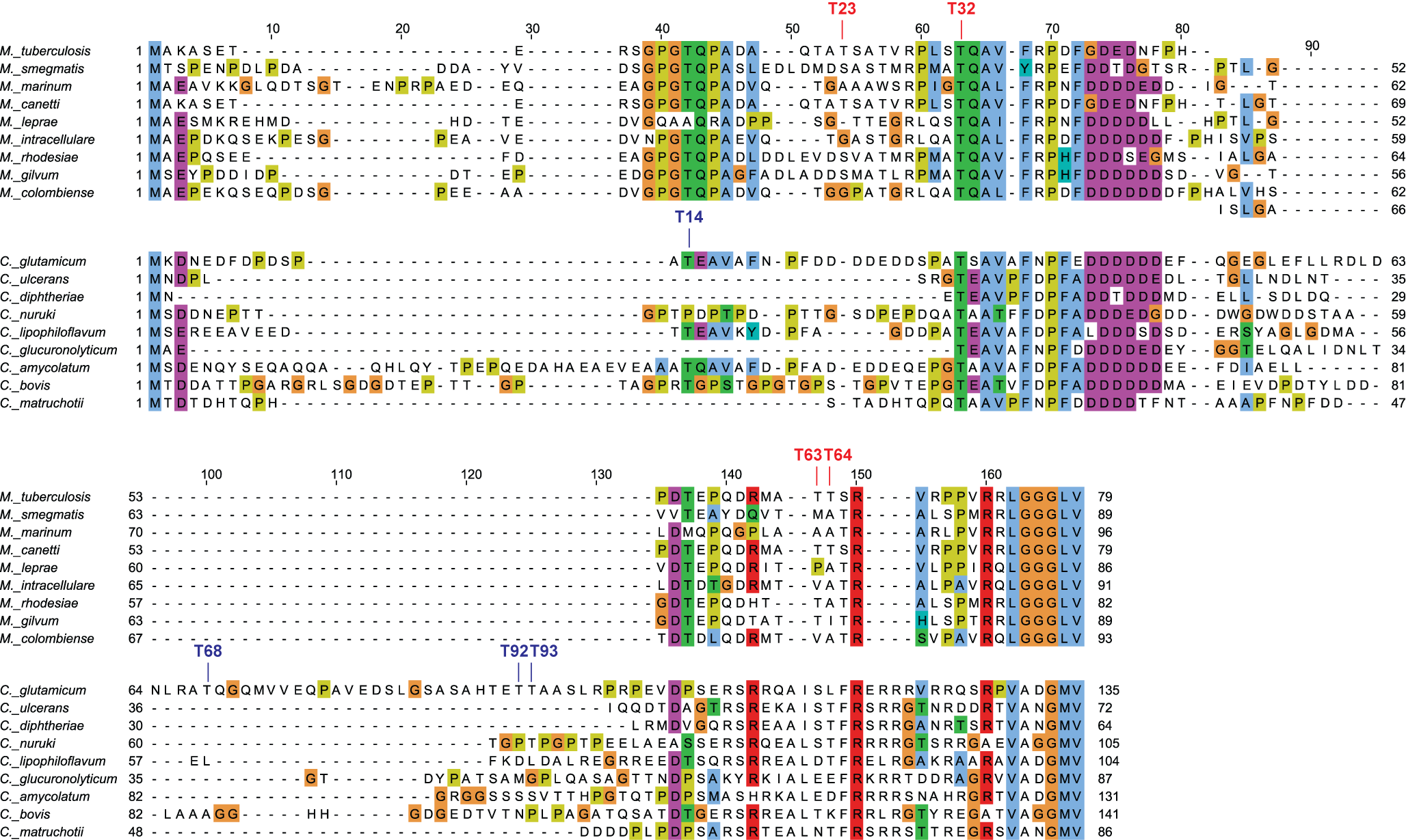

Supplement: FIG S3 [file mbio.01717-21-sf003.tif]

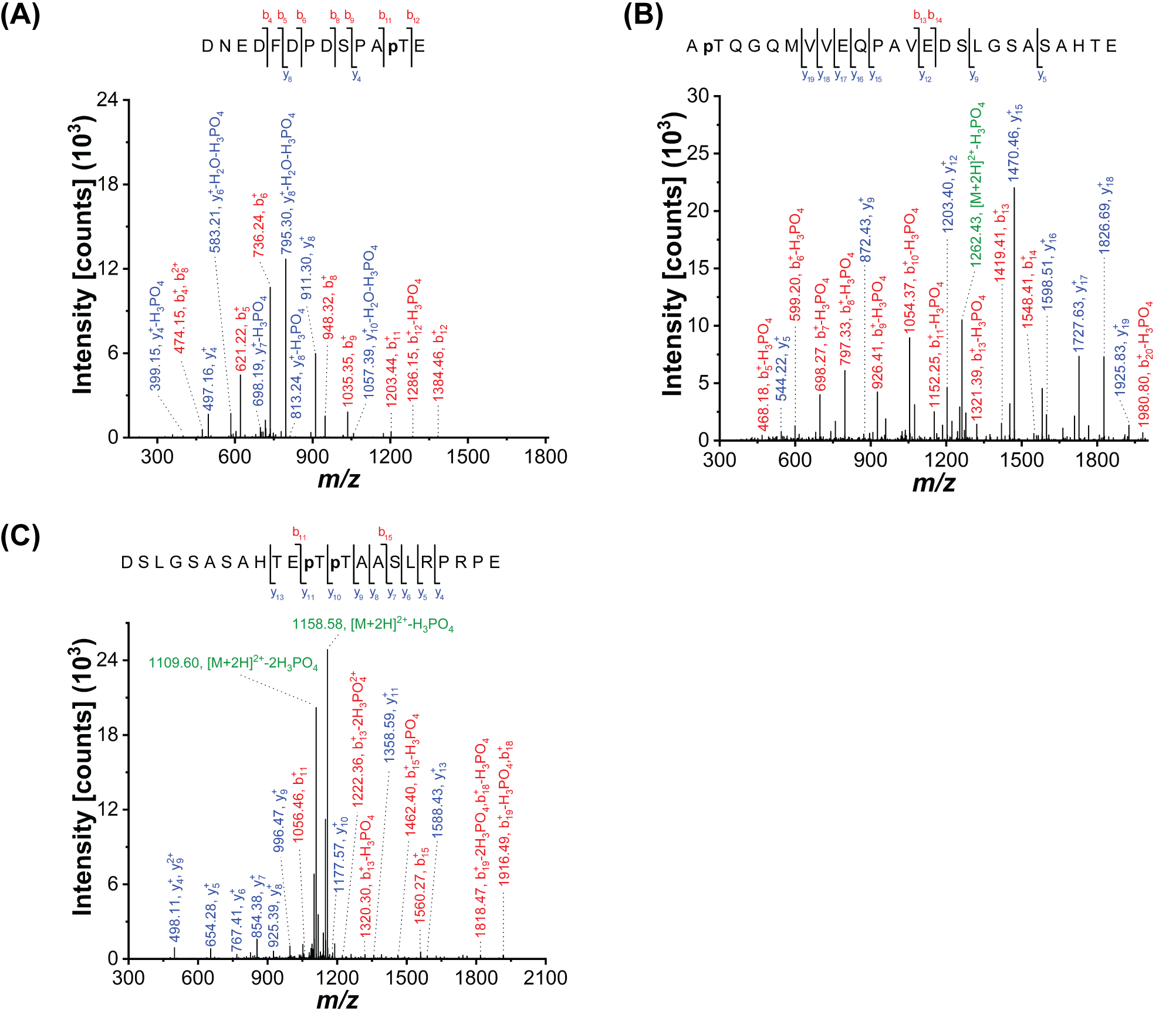

Supplement: FIG S4 [file mbio.01717-21-sf004.tif]

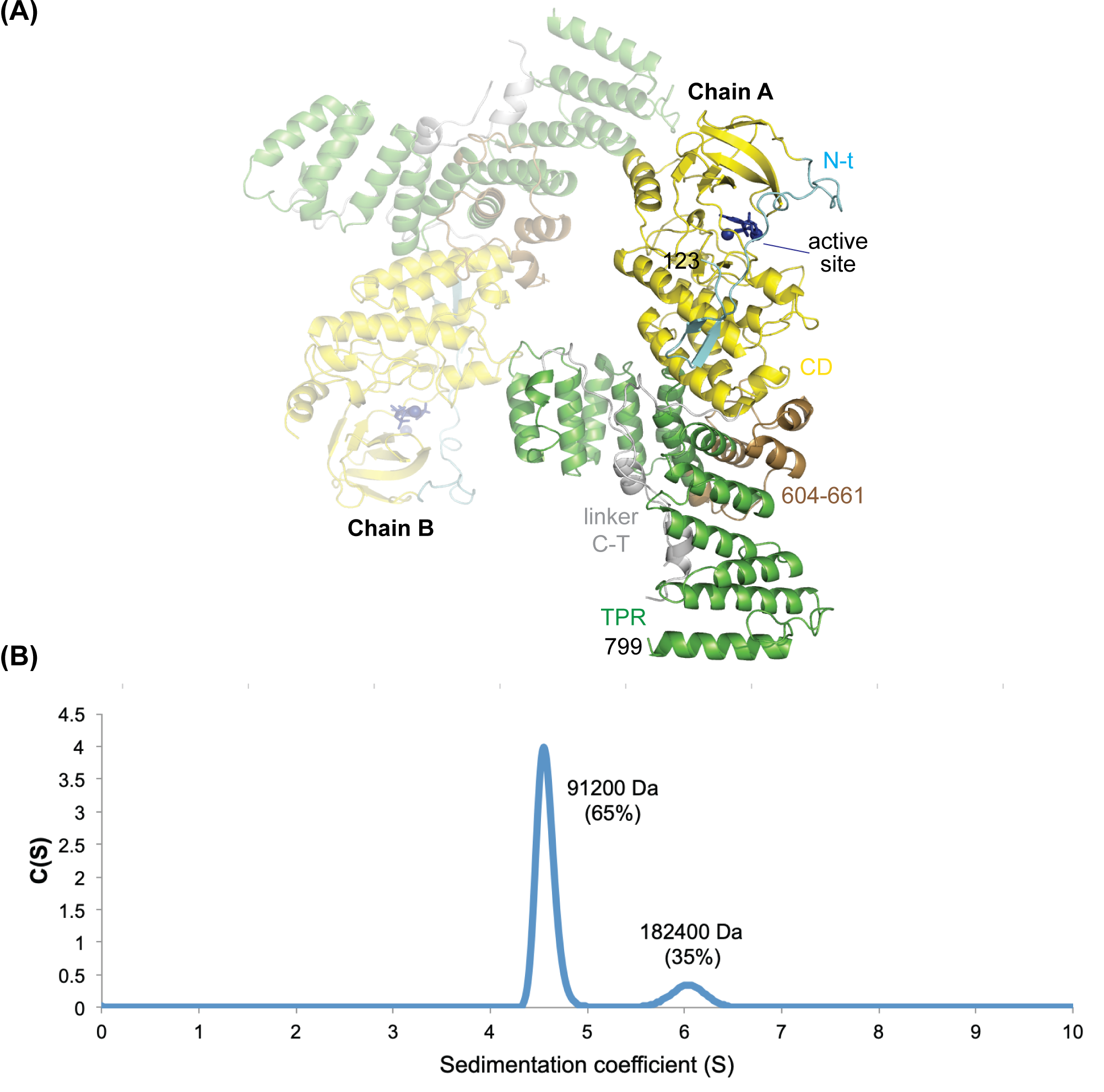

Supplement: FIG S5 [file mbio.01717-21-sf005.tif]

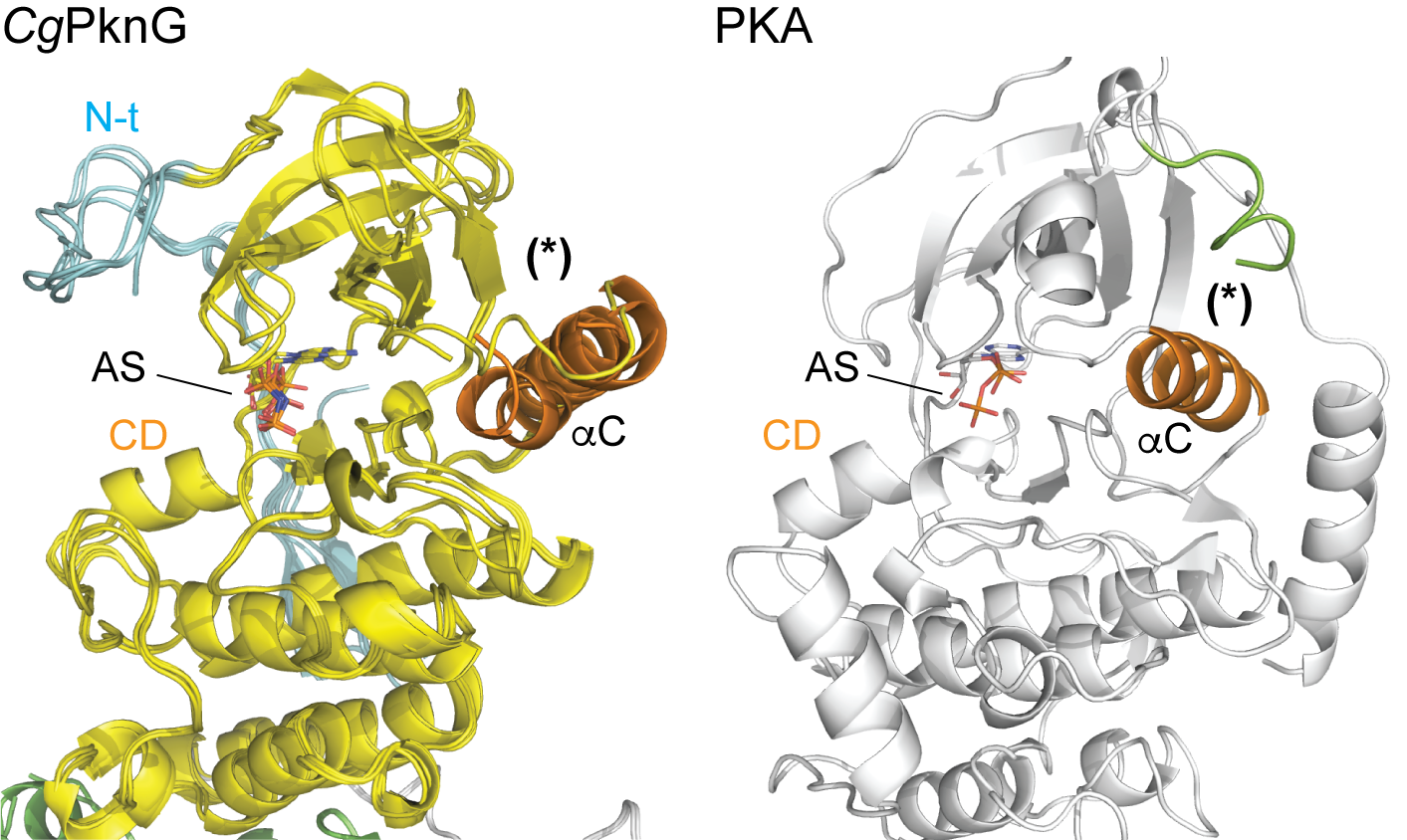

Supplement: FIG S6 [file mbio.01717-21-sf006.tif]

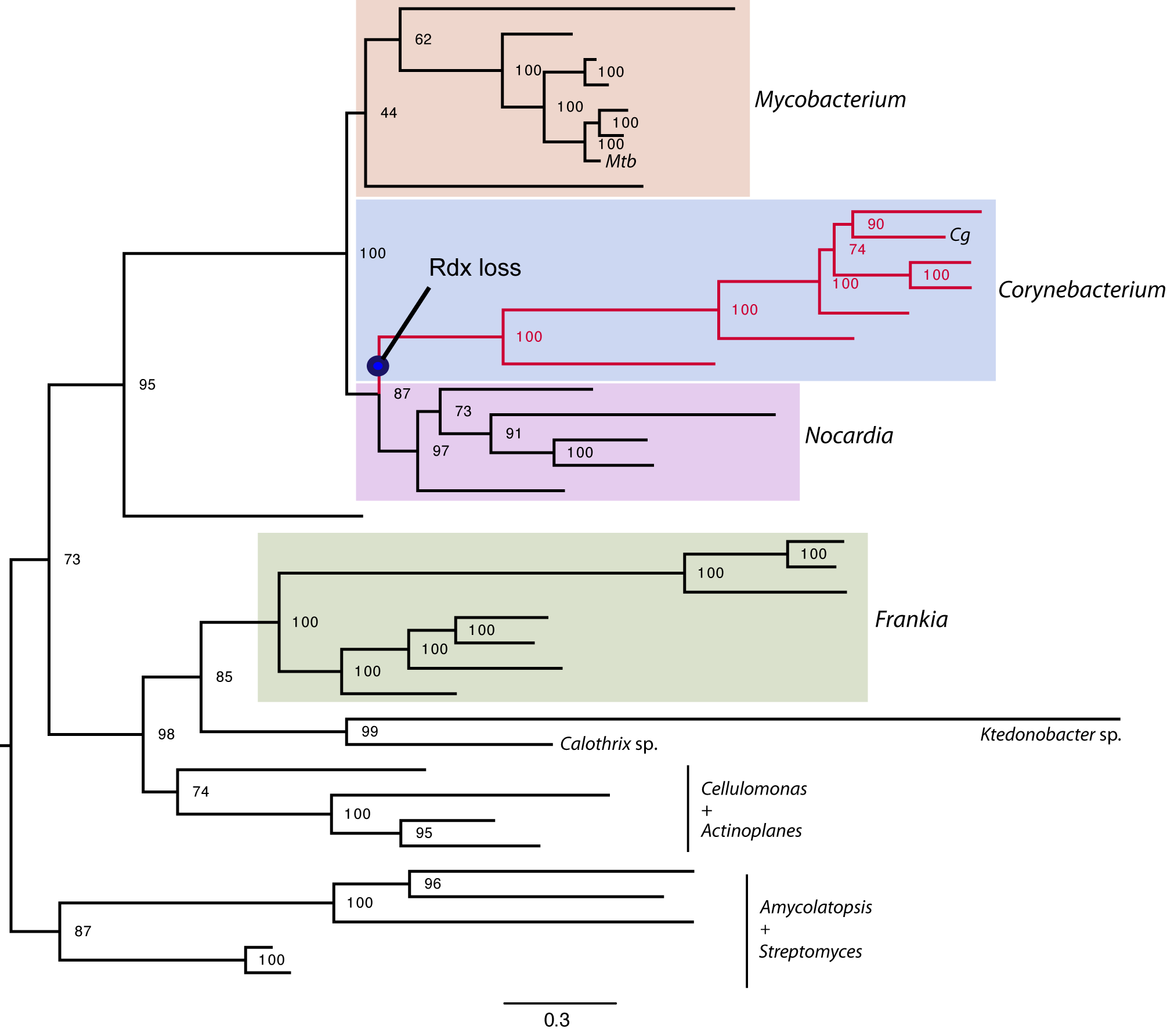

Supplement: FIG S7 [file mbio.01717-21-sf007.tif]
